# Maladaptive Eating Behaviours, Generalised Anxiety Disorder and Depression Severity: A Comparative Study between Adult Women with Overweight, Obesity, and Normal Body Mass Index Range

**DOI:** 10.3390/nu16010080

**Published:** 2023-12-26

**Authors:** Tomasz Witaszek, Mateusz Babicki, Anna Brytek-Matera, Agnieszka Mastalerz-Migas, Krzysztof Kujawa, Karolina Kłoda

**Affiliations:** 1 Hipokrates Przychodnia Lekarzy Rodzinnych i Specjalistów, ul. Powstańców WIelkopolskich 1a, 64-330 Opalenica, Poland; witaszek.tomasz@gmail.com; 2 Department of Family Medicine, Wroclaw Medical University, 50-367 Wrocław, Poland; agnieszka.mastalerz-migas@umw.edu.pl; 3 Eating Behavior Laboratory (EAT Lab), Institute of Psychology, University of Wroclaw, ul. J. Dawida 1, 50-527 Wrocław, Poland; anna.brytek-matera@uwr.edu.pl; 4 Statistical Analysis Centre, Wroclaw Medical University, 50-368 Wrocław, Poland; 5 MEDFIT Karolina Kłoda, ul. Narutowicza 13E/11, 70-240 Szczecin, Poland; wikarla@gazeta.pl

**Keywords:** obesity, maladaptive eating behaviours, anxiety, depression, women health, BMI

## Abstract

(1) Background: Causes of obesity are multifactorial and include genetic predisposition as well as behavioural, psychological, social, and hormonal influences. We aimed to compare adult women with normal weight, overweight, and obesity, with a focus on maladaptive eating behaviours, the presence of generalised anxiety disorder, and the severity of depression. Additionally, we explored the context of anti-obesity pharmacotherapy and the status of bariatric surgery. (2) Methods: The sample was composed of 1105 adult women. The following measures, through the Computer-Assisted Web Interview (CAWI), were used in the present study: the Three Factor Eating Questionnaire (TFEQ-R18), the 7-item Generalised Anxiety Disorders Scale (GAD-7), and the 9-item Patient Health Questionnaire (PHQ-9). (3) Results: All domains of the TFEQ-R18 had correlations with Body Mass Index (BMI). There was a weak negative association between BMI and Cognitive Restraint (r = −0.172, *p* < 0.001) and a weak positive relationship between BMI and Uncontrolled as well as Emotional Eating (r = 0.165, *p* < 0.001; r = 0.191, *p* < 0.001, respectively). Women who screened positive for anxiety scored lower in the Cognitive Restraint domain (10.11 ± 3.58, *p* = 0.042) and higher in the Uncontrolled Eating (12.69 ± 6.04, *p* < 0.001) and Emotional Eating (5.29 ± 2.75, *p* < 0.001) domains. Similarly, women screening positive for depression had lower scores in Cognitive Restraint (9.88 ± 3.61, *p* < 0.001) and higher scores in Uncontrolled Eating (12.64 ± 6.09, *p* < 0.001) and Emotional Eating (5.31 ± 2.71, *p* < 0.001). A significant association between liraglutide and semaglutide administration and Cognitive Restraint was observed. (4) Conclusions: Individualised treatment for obesity should consider the existing and confirmed association between maladaptive eating behaviours and generalised anxiety disorder, as well as the severity of depression influencing the BMI altogether. The use of anti-obesity pharmacotherapy needs further exploration because the evidence for the use of liraglutide and semaglutide in terms of positive associations with eating behaviours is encouraging.

## 1. Introduction

Since 1975, there has been a threefold increase in obesity and overweight prevalence. In 2019, an estimated 38.2 million children under the age of 5 were overweight or obese, which is an unfavourable prognosis for the future. It is estimated that around 55% of children with obesity will become adolescents with obesity, and out of them, around 80% will become adults living with this disease [1,2]. Obesity is the result of complex relationships between epigenetic, genetic, psychological, socioeconomic, and cultural factors [3,4]. Due to its common occurrence, obesity is now an emerging topic of scientific study, not only in terms of pathogenesis but also because of its economic and societal impact [5]. Positive energy balance in obesity is related to numerous factors, including eating patterns affected by maladaptive eating behaviours or potentially occurring eating disorders. In the literature, three specific dimensions of eating behaviour are presented: cognitive restraint (placing restrictions on food without taking into account the sensations of hunger and satiety), uncontrolled eating (characterised by a disordered perception of hunger that results in excessive food intake), and emotional eating (where food is consumed in response to various negative emotions) [4,6]. The Three Factor Eating Questionnaire (TFEQ) is a widely used method that quantifies these three dimensions [7,8]. In the case of the Polish population, it was found that the three-factor structure of the TFEQ-R18 (18-items) is invariant at each level–configural, metric, scalar, and strict [6]. Several studies have examined the associations between body weight and TFEQ-R18 scores. They demonstrated that gaining weight is significantly associated with uncontrolled eating and emotional eating and that higher scores in cognitive restraint and emotional eating are found to be associated with a higher Body Mass Index (BMI) in women [9,10,11].

Apart from the fact that psychological factors influence the development of obesity, this disease is also the cause of mental disorders. Anxiety and depression have a high prevalence among people with obesity, especially females [12]. According to a recent meta-analysis by Jung et al. (2017), obesity increases the risk of depression [13]. A bidirectional link between these two diseases has been observed [14]. Individuals with more severe obesity (BMI ≥ 40 kg/m^2^) exhibited a stronger association with depression [13,14]. In some studies, the link between being overweight and the onset of depression was found in women but not in men [13]. For women with a baseline BMI of 30 kg/m^2^ or higher, the odds of a major depressive disorder (MDD) are significantly higher and independent of other risk factors such as age, education, prior depressive symptoms, marital status, chronic disease, low social support, and financial strain [15]. A connection was also found between obesity and general anxiety disorder (GAD), but the risk between obesity and the onset of anxiety disorders (i.e., panic disorder, agoraphobia without panic disorder, social phobia, specific phobia, and generalised anxiety disorder) has been less investigated so far [16]. Women who were either overweight or obese were significantly more likely to have the three mental health issues simultaneously—current depression; lifetime diagnosed depression; and anxiety disorder—than women with a normal BMI range [17].

Achieving long-term weight loss maintenance in patients with obesity through current treatment methods continues to pose a clinical challenge. This challenge is particularly evident in the varied responses to obesity interventions, including changes in eating patterns, medication use, and surgical procedures [4]. Lifestyle interventions for weight loss are currently not personalised to address the underlying pathophysiology and behavioural traits associated with obesity [18]. There is a need to develop a valid classification for this heterogeneous patient population and assess its effectiveness in obesity management to provide a more individualised approach. One of the hypothesised obesity phenotypes is emotional eating, which could be identified by screening patients for depression, anxiety, and emotional eating behaviour using validated tools [4,18]. This approach is especially promising because a phenotype-targeted pharmacological treatment of obesity resulted in a mean weight loss of 4.1% higher after 6 months compared to the non-phenotype-targeted approach. Additionally, the proportion of patients who lost >10% at 1 year was 79%, compared with 34% with non-phenotype-guided treatment, with no increase in the adverse effect ratio [4].

The aim of the present study was to compare adult women with normal weight, overweight, and obesity in relation to maladaptive eating behaviours, generalised anxiety disorder, and depression severity using the TFEQ-R18, the 7-item Generalised Anxiety Disorders Scale (GAD-7) and the 9-item Patient Health Questionnaire (PHQ-9). As this topic is severely understudied, we have also explored the associations between anti-obesity pharmacotherapy and undergoing bariatric surgery in the past with TFEQ-R18 domain scores. We have put forward the following hypotheses: 1. There is an association between BMI and the results of the above-mentioned questionnaires, and the significance of this relationship will increase with weight. 2. There is a correlation between the TFEQ-R18 scales and the PHQ-9 and GAD-7. 3. The use of particular medications influences the scores obtained on the subscales of the TFEQ-R18 scale.

## 2. Materials and Methods

### 2.1. Participants and Recruitment

This cross-sectional survey was conducted between 4 September 2023 and 19 October 2023 using a computer-assisted web interviewing (CAWI) technique. A proprietary questionnaire was used and uploaded to Google Forms, making it available to any device connected to the internet. The survey was then distributed through social media platforms like Instagram and Facebook within support groups for individuals with obesity, undergoing pharmacological obesity treatment, or undergoing bariatric surgery. Before proceeding with the questionnaire, participants were informed about the research objectives and methodology, after which they gave their informed consent to participate in this study. Respondents had the option to discontinue their participation without explanation at any point. In order to maintain anonymity, no personal data were collected. This study’s inclusion criteria were being female, being over 18 years old, residing in Poland, and having internet access.

This study was conducted in accordance with the Declaration of Helsinki and approved by the Bioethics Committee of the Wroclaw Medical University, Poland (approval number: 349/2023N).

This study questionnaire included a series of questions aimed at evaluating the socio-demographic profile of the participants. These questions covered topics such as age, gender, height, current weight, and the highest weight ever recorded. Following this section, participants were asked about the presence of any chronic diseases, whether they were undergoing pharmacological treatment for obesity, or if they had undergone bariatric treatment. In the event of a positive response, participants were presented with an additional multiple-choice question to specify the particular co-morbid condition, specific anti-obesity medication taken, and type of bariatric procedure. The second part of the survey consisted of standardised psychometric tools: the Three Factor Eating Questionnaire (TFEQ-R18), the Patient Health Questionnaire (PHQ-9), and the General Anxiety Disorder 7-Item (GAD-7). The questionnaire required all questions to be answered. Failure to answer at least one question prevented progression to the next stage.

### 2.2. TFEQ-R18 Scale

The Three Factor Eating Questionnaire (TFEQ-R18) was developed by Stunkard and Messick [7] as a 51-item questionnaire. It was further modified by Karlsson et al. [19], who abbreviated the measure to 18 items (TFEQ-R18). The Polish version of the TFEQ-R18 was validated [6]. The questionnaire refers to current dietary practise and measures three domains of eating behaviour: cognitive restraint (conscious restriction of food intake in order to control body weight), uncontrolled eating (excessive food intake due to a loss of control accompanied by subjective feelings of hunger), and emotional eating (inability to resist emotional cues). The TFEQ-R18 consists of 17 items on a four-point Likert response scale (ranging from “definitely true” to “definitely false”) and an additional item to rate, on an eight-point Likert scale, how often respondents restrain their eating. For the purpose of analysis, this particular item was re-coded into four categories, with responses 1 and 2 combined into category 1, 3 and 4 into category 2, 5 and 6 into category 3, and 7 and 8 into category 4. The range of points that can be obtained in each subscale is: 0–18 for cognitive restraint, 0–27 for uncontrolled eating, and 0–9 for emotional eating. The reliability of the tool, as measured by Cronbach’s alpha coefficient, was 0.763. Additionally, the Cronbach’s alpha coefficient for the individual subscales was, respectively, 0.799 for cognitive restraint, 0.889 for uncontrolled eating, and 0.883 for uncontrolled eating.

### 2.3. PHQ-9 Scale

The Patient Health Questionnaire (PHQ-9) is an instrument used to diagnose criteria-based depressive disorders commonly encountered in primary care settings. The PHQ-9 asks patients to rate, on a four-point scale ranging from “not at all” to “most days”, the frequency with which they have experienced certain depressive symptoms in the preceding 2 weeks. Items are rated from 0 to 3 accordingly. The possible point range to be scored is between 0 and 27 points. Researchers have confirmed the validity and reliability of the PHQ-9 [20]. The Polish version of the questionnaire has been validated [21]. In the validation of the tool in the Polish population, a cutoff value of 12 points was calculated [21]. The reliability of the tool, as measured by Cronbach’s alpha coefficient, was 0.872.

### 2.4. GAD-7 Scale

The General Anxiety Disorder 7-Item (GAD-7) Scale is commonly used to assess general anxiety symptoms in various settings and populations. The GAD-7 has shown good reliability and construct validity [22]. It comprises seven items that measure worry and anxiety symptoms. Each item is rated on a four-point Likert scale (0–3), with total scores ranging from 0 to 21, where higher scores indicate greater anxiety severity. Scores exceeding 10 are considered to be within the clinical range. In this study, we used the Polish translation of the GAD-7 provided by the MAPI Research Institute [23]. A high internal consistency for Cronbach’s alpha was found (0.924).

For a better understanding of the research methodology, the English version of the survey is attached in Supplementary Table S1. The English Version of this Study Questionnaire.

### 2.5. Statistical Analysis

The variables in this study had both qualitative and quantitative characteristics. To assess the normality of the distribution, the Shapiro–Wilk test was used. Qualitative variable comparisons were conducted using the chi-squared test. For quantitative variables, non-parametric tests such as the Kruskal–Wallis H Test or the Mann–Whitney U test were used. The level of correlation between variables was assessed with the Spearman correlation test coefficient. The BMI was assumed to mediate the effect of eating behaviour (TFEQ-R18 subscales) on anxiety (GAD-7) and depression (PHQ-9), because it can be affected by the eating behavior, and on the other hand, it can affect anxiety or health. Based on this assumption, the associations between eating behavior, BMI, anxiety, and health were analysed using the mediation model. The model enables assessing the average direct effect (ADE) of a given factor (subscale of TFEQ-R18) and the average causal mediation effect (ACME) of a mediator (BMI). To test hypothesis 1, the Kruskal–Wallis H Test and Mann–Whitney U test were used. Additionally, the mediation model was used. To test hypothesis 2, the Spearman correlation test was used, and to test hypothesis 3, the Mann–Whitney U test was used.

The hypotheses verified based on a common group of patients were considered a family of hypotheses, and the Bonferroni correction was used for controlling I-type error. Therefore, a corrected alpha level amounted to 0.008 (0.05/6) (with respect to scales GAD-7 and PHQ-9), or to 0.017 (0.05/3) (for Age and BMI). The analysis was conducted using Statistica 13.0 (TIBCO Software, Palo Alto, CA, USA). The mediation analysis was performed using the command ‘mediate’ from the R-package version 4.0.4 ‘mediation’.

## 3. Results

### 3.1. Characteristics of the Study Group

This study involved 1105 female participants. The average age was 38.89 years (SD = 9.0). The mean BMI was 33.32 kg/m^2^ (SD = 6.67). Overall, 44.2% of women (*n* = 488) were undergoing pharmacological treatment for obesity, with liraglutide being the most common (21.3%), and 11.9% of women (*n* = 131) had undergone surgical treatment for obesity. Among all participants, 29.6% had one or more chronic diseases. Out of 68.7% of women with obesity (*n* = 759), only 44.8% (*n* = 496) recognised it as a chronic disease. Approximately 46.4% of the women (*n* = 513) screened positively for anxiety, and 50.3% (*n* = 556) screened positively for depression. A detailed overview of the characteristics of the study group is presented in Table 1, and a summary of the results and interpretation of the Three-Factor Eating Questionnaire, Generalised Anxiety Disorder, and Patient Health Questionnaire-9 is presented in Table 2.

### 3.2. Correlations between the Three Domains of TFEQ-R18 and GAD-7, as Well as PHQ-9 Scores in Women with Normal Weight, Overweight, and Obesity

All domains of TFEQ-R18 were associated with BMI. There were weak negative correlations between BMI and Cognitive Restraint, (rho = −0.172, *p* < 0.001) and weak positive relationships between BMI and Uncontrolled as well as Emotional Eating (rho = 0.165, *p* < 0.001; rho = 0.191, *p* < 0.001, respectively). Emotional Eating was the only domain that was weakly negatively linked to age (rho = −0.086, *p* = 0.004).

Analysis of correlations between three domains of TFEQ-R18 and GAD-7 as well as PHQ-9 scores (as continuous values) revealed no significance for GAD-7 and PHQ-9 between Uncontrolled and Emotional Eating in women with normal weight. In overweight women, relationships were moderately significantly positive for both the GAD-7 and PHQ-9 scores in relation to Uncontrolled Eating (rho = 0.357, *p* < 0.001, and rho = 0.360, *p* < 0.001, respectively) and Emotional Eating (rho = 0.313, *p* < 0.001, and rho = 0.349, *p* < 0.001, respectively). Finally, we found weak negative significant correlations among women with class I obesity between GAD-7 and PHQ-9 scores and Cognitive Restraint (rho = −0.136, *p* = 0.008 and rho = −0.189, *p* < 0.001, respectively) and weak positive relationship between GAD-7 and PHQ-(score and Uncontrolled Eating (rho = 0.283, *p* < 0.001 and rho = 0.291 and *p* < 0.001, respectively) and Emotional Eating (rho = 0.286, *p* < 0.001 and rho = 0.314, *p* < 0.001, respectively). All analysed correlations are presented in Table 3.

Associations between the three domains of TFEQ-R18 and GAD-7, as well as PHQ-9 scores (grouped by the screening cut-off threshold) revealed that women who screened positively for anxiety were scoring higher in the Uncontrolled Eating (12.69 ± 6.04, *p* < 0.001) and Emotional Eating (5.29 ± 2.75, *p* < 0.001) domains when compared to those that did not meet the screening threshold.

A similar tendency was noted for women who screened positively for depression. They were scoring lower in the Cognitive Restraint domain (9.88 ± 3.61, *p* < 0.001) while scoring higher in the Uncontrolled Eating (12.64 ± 6.09, *p* < 0.001) and Emotional Eating (5.31 ± 2.71, *p* < 0.001) domains.

After dividing the study group into BMI subgroups, it was found that women with normal weight presented no association between GAD-7 and PHQ-9 scores and TFEQ-R18 Cognitive Restraint scores, whereas in women with class I obesity, an association between the PHQ-9 score and Cognitive Restraint was observed. Moreover, in women with class I and class III obesity, the PHQ-9 score was significantly associated with all three domains of TFEQ-R18. A detailed overview of the results of GAD-7 and PHQ-9 is presented in Table 4, distinguishing between different BMI subgroups.

### 3.3. Maladaptive Eating Behaviours among Adult Women with Overweight, Obesity, and Normal Weight

After dividing participants into subgroups based on their BMI, we found an association between BMI and all three TFEQ-R18 domains. We observed increasing scores of Uncontrolled and Emotional Eating and decreasing scores of Cognitive Restraint in women with an increasing degree of obesity (*p* < 0.001).

Patients who are undergoing pharmacological treatment for obesity scored significantly higher in the Cognitive Restraint domain (*p* < 0.001). When considering every class of medication separately, this relationship was stronger with semaglutide (*p* < 0.001) than with liraglutide (*p* < 0.016). There was no observed relationship between cognitive restraint and the use of Bupropion/Naltrexone. However, it is the only medication for which a relationship with Emotional Eating was noted (*p* < 0.001). The status of bariatric surgery had significant differences in all three domains (*p* < 0.001). An in-depth overview of the results of the TFEQ-R18 is presented in Table 5.

### 3.4. Mediation Effect of BMI in Associations between the TFEQ-R18 and Anxiety and Health

Although the mediation effect of BMI was statistically significant in all the analyses performed, the effect size of the mediation was low. The rate of the mediated effect on the total effect of the analysed factors amounted to 0.04–0.06 (4–6%) for uncontrolled eating and emotional eating and to 0.20 and 0.16 (20 and 16%) for cognitive restraint (Table 6).

## 4. Discussion

The aim of this study was to compare women with normal body weight, overweight, and obesity in relation to maladaptive eating behaviours, generalised anxiety disorder, and depression severity using the TFEQ-R18, the GAD-7, and the PHQ-9 scales. We have also hypothesised that there will be an association between body weight and scores on the aforementioned questionnaires and that the significance of this association will increase with increasing BMI.

Indeed, we found that BMI correlated significantly positively with Uncontrolled and Emotional Eating and significantly negatively with Cognitive Restraint. This was confirmed in a separate analysis of associations between the studied variables and TFEQ-R18 domain scores. An analysis of existing literature showed that this relationship may differ in relation to the studied population. For example, in the Finnish study on 2 997 females, higher BMI was associated with higher levels of Cognitive Restraint (*p* < 0.001) and Emotional Eating (*p* < 0.001), but not with Uncontrolled Eating [10]. Whereas, in the Arabic population, BMI was positively correlated with Uncontrolled Eating and Emotional Eating (*p* < 0.001 respectively), but not with Cognitive Restraint [8]. Another piece of evidence that this study population is of great importance gives a sample of 4377 Swedish, middle-aged men and women living with obesity in whom the original factor structure of TFEQ was not replicated at all [19]. Not without significance is also the mean age of the studied population, because it can be correlated with TFEQ-R18 domain scores. In the case of our study, a significant negative correlation was found between age and Emotional Eating. The aforementioned Finnish population consisted of very young women, with a mean age of 18.6 years, whereas our population consisted of mostly middle-aged women, with a mean age of 38.9 years. Our results also vary in the context of Cognitive Restraint, which is usually associated positively with a higher BMI [10,24] or is not associated with body weight [5,25]. Interestingly, a German sample of middle-aged adults exhibited a reversed U-shaped association between cognitive restraint and BMI, where BMI was high in subjects with medium cognitive restraint [24]. Population diversity in terms of the association between BMI and TFEQ-R18 is an interesting aspect that requires further research.

Our study revealed significant correlations between PHQ-9 scores and all three domains of TFEQ-R18. Also, when dividing the population into groups by BMI, relationships were noted for Uncontrolled Eating and Emotional Eating across all BMI subgroups. A similar observation was made in a study of 238 participants undergoing a behavioural weight loss intervention in Germany. PHQ-9 scores were positively correlated with Hunger (Emotional Eating) (r = 0.245, *p* < 0.001) and Disinhibition (Uncontrolled Eating) (r = 0.353, *p* < 0.05). It is worth noting that the original version of the 51-item TFEQ was used. No significant correlation was observed between PHQ-9 scores and Cognitive Restraint [26]. Individuals with obesity and eating disorders are characterised by impulsivity, anxiety, and depression. However, even without the presence of eating disorders, people living with obesity are affected by psychological factors [27,28]. The linkage between weight gain and depression is considered to be formed on the basis of pathological eating behaviour, including emotional eating [29]. Thus, the results observed in the 1105 population of women in this study are in concordance with former reports and confirm the previous scientific findings.

The aforementioned study of the German population undergoing behavioural weight loss intervention also reveals correlations between GAD-7 scores and TFEQ-R18 Domains. GAD-7 scores are positively correlated with Hunger (Emotional Eating) (r = 0.311, *p* < 0.001) and Disinhibition (Uncontrolled Eating) (r = 0.245, *p* < 0.001) [26]. Another study on 129 Ghanaian students reveals a positive correlation between screening positively for anxiety with GAD-7 and Emotional Eating (r = 0.471, *p* < 0.001), but only for female participants [30]. Similar to depression, anxiety, and perceived stress affect individuals with obesity. Especially, perceived stress is considered a linkage between body image, stigma, and depressive symptoms, as well as food addiction [5,31,32,33]. There is an association between weight management and stress management. Optimal stress coping strategies result in weight reduction and changes in pathologic eating behaviours in women [34].

In one of the studies, TFEQ-R18 scores were measured before and after liraglutide treatment. After treatment with liraglutide, the Uncontrolled Eating score decreased from 36.8 ± 24.5 to 19.6 ± 18.4 (*p* < 0.001), and the Emotional Eating score decreased from 49.9 ± 33.3 to 28.5 ± 26.9 (*p* < 0.001). Scores for Cognitive Restraint were not changed [35]. What is interesting is that in our study, a significant association between liraglutide administration and Cognitive Restraint was observed. We can assume that participants already treated with liraglutide (before the study) had undergone lifestyle interventions and education, which are provided with obesity pharmacotherapy in Poland, thus their Cognitive Restraint obtained higher scores. A similar result was observed in the case of semaglutide.

We also found a relationship between the use of Bupropion/Naltrexone and the Emotional Eating domain of TFEQ-R18 (*p* < 0.001). However, it should be suspected that the presence of emotional eating is the reason why the participant received this medication in the first place. The Polish Society for the Treatment of Obesity recommends considering the use of this medication in patients exhibiting emotional eating patterns and significant cravings, especially with coexisting depression [36]. In one of the studies about phenotype-targeted pharmacological treatment, the dominance of the emotional eating domain in TFEQ was an indication for choosing this particular class of anti-obesity medication and led to a higher efficacy of treatment [4].

The status of bariatric surgery had significant differences in all three domains of TFEQ-R18 (*p* < 0.001). Those who underwent the surgery scored higher in Cognitive Restraint, while receiving lower scores in Emotional Eating and Uncontrolled Eating. This is reflected in the available literature. In one of the studies, 204 adults with severe obesity from three countries were followed 1 year after metabolic surgical procedures. The original version of the 51-item TFEQ was used. After 12 months, there were statistically significant increases in restraint and decreases in disinhibition and hunger [37].

The authors are aware of the limitations of this study, which are undoubtedly the data collection methodology. Firstly, the authors did not analyse socio-economic data, which can play a very large role in the parameters analysed. Furthermore, the use of self-reported height and weight to calculate BMI is controversial, as there are numerous studies that suggest self-reported measures are biassed as people tend to provide overestimates of their height and underestimates of their weight [38,39]. Additionally, the use of an online questionnaire and its distribution through social networks carry a risk of group selection. Only people using the Internet and social networks could participate in this study. However, on the other hand, this type of methodology allows a large number of people from different parts of the country to be reached in a quick way. In addition, research suggests that CAWI-type surveys are associated with a greater likelihood of providing truthful answers than socially acceptable ones. Participants also experience lower levels of stress when taking part in this type of survey. Another limitation of the survey is the lack of representativeness of the analysed group in Polish society. It should also be borne in mind that the authors do not know the number of people reached by the survey and cannot assess the response rate. More research on eating-related behaviours among patients is needed to understand the multifaceted nature of these disorders and to offer new solutions with a more individual approach.

## 5. Conclusions

Individualised treatment for obesity should consider the existing and confirmed association between maladaptive eating behaviours, and generalised anxiety disorder, as well as the severity of depression influencing the BMI altogether. The use of anti-obesity pharmacotherapy needs further exploration because the evidence for the use of liraglutide and semaglutide in terms of positive associations with eating behaviours is encouraging.

## Figures and Tables

**Table 1:** Characteristics of the study group.

Variable		N/M ± SD
Age [years]		38.89 ± 9.00
Body Mass Index [kg/m^2^]		33.32 ± 6.67
Body Mass Index	Underweight	0 (0.0)
	Normal weight	92 (8.3)
	Overweight	254 (23.0)
	Obesity I	366 (33.1)
	Obesity II	247 (22.4)
	Obesity III	146 (13.2)
Pharmacological treatment of obesity		488 (44.2)
Pharmacological treatment of obesity	Semaglutide	232 (21.0)
	Bupropion + Naltrexone	41 (3.7)
	Liraglutide	235 (21.3)
Surgical treatment of obesity		131 (11.9)
Chronic diseases		327 (29.6)
Chronic diseases	Hypertension	220 (19.9)
	Cardiovascular diseases other than hypertension	57 (5.2)
	Obesity	496 (44.8)
	Hypothyroidism	387 (35.0)
	Diabetes mellitus type 2	116 (10.5)
	Osteoarthritis	76 (6.9)
	Depression	211 (19.1)
	Anxiety	153 (13.8)
	Dyslipidemia	101 (9.1)
	Fatty liver disease	112 (10.1)
	Other	367 (33.2)

M—mean; SD—Standard deviation; N—number,, kg/m^2^—kilogram(s) per square metre.

**Table 2:** Summary of results and interpretation of the Three-Factor Eating Questionnaire, Generalised Anxiety Disorder, and Patient Health Questionnaire-9.

Variable		N/M ± SD
TFEQ-R18	Cognitive Restraint	10.33 ± 3.51
	Uncontrolled Eating	11.16 ± 5.85
	Emotional Eating	4.60 ± 2.76
GAD-7		9.57 ± 6.10
GAD-7 Interpretation	Anxiety	513 (46.4)
PHQ-9		10.6 ± 6.36
PHQ-9 Interpretation	Depression	556 (50.3)

M—mean; SD—Standard deviation; N—number; TFEQ-R18 Three-Factor Eating Questionnaire, GAD-7—Generalised anxiety disorder; PHQ-9—Patient Health Questionnaire-9.

**Table 3:** Correlations between the three domains of TFEQ-R18, GAD-7, and PHQ-9 scores (as a continuous value) in different BMI subgroups.

Variable	Cognitive Restraint		Uncontrolled Eating		Emotional Eating	
	rho	*p* *	rho	*p* *	rho	*p* *
Age [years]	0.055	0.067	−0.428	0.154	−0.086	**0.004**
BMI [kg/m^2^]	−0.172	**<0.001**	0.165	**<0.001**	0.191	**<0.001**
Whole group (N = 1105)						
GAD-7	−0.091	**0.003**	0.299	**<0.001**	0.293	**<0.001**
PHQ-9	−0.154	**<0.001**	0.338	**<0.001**	0.329	**<0.001**
Normal weight (N = 92)						
GAD-7	0.048	0.648	0.179	0.086	0.149	0.155
PHQ-9	0.008	0.937	0.268	**0.008**	0.208	0.046
Overweight (N = 254)						
GAD-7	−0.040	0.524	0.357	**<0.001**	0.313	**<0.001**
PHQ-9	−0.112	0.074	0.360	**<0.001**	0.349	**<0.001**
Obesity I (N = 366)						
GAD-7	−0.136	**0.008**	0.283	**<0.001**	0.286	**<0.001**
PHQ-9	−0.189	**<0.001**	0.291	**<0.001**	0.314	**<0.001**
Obesity II (N = 247)						
GAD-7	−0.002	0.981	0.221	**<0.001**	0.215	**<0.001**
PHQ-9	0.005	0.934	0.207	**0.002**	0.191	**0.003**
Obesity III (N = 146)						
GAD-7	−0.118	0.156	0.291	**<0.001**	0.318	**<0.001**
PHQ-9	−0.316	**<0.001**	0.473	**<0.001**	0.435	**<0.001**

N—number; GAD-7—Generalised anxiety disorder; PHQ-9—Patient Health Questionnaire-9; * Spearman’s rank correlation. Significant effects (<0.017 for BMI and age) and (<0.008) for the rest are marked in bold.

**Table 4:** Association between the three domains of TFEQ-R18, GAD-7, and PHQ-9 scores (grouped by the screening cut-off threshold).

Variable			Cognitive Restraint		Uncontrolled Eating		Emotional Eating	
			M ± SD	*p* *	M ± SD	*p* *	M ± SD	*p* *
Whole group (N = 1105)								
GAD-7 Interpretation	Anxiety		10.11 ± 3.58	0.042	12.69 ± 6.04	**<0.001**	5.29 ± 2.75	**<0.001**
	No anxiety		10.53 ± 3.43		9.82 ± 5.32		3.99 ± 2.61	
PHQ-9 Interpretation	Depression		9.88 ± 3.61	**<0.001**	12.64 ± 6.09	**<0.001**	5.31 ± 2.71	**<0.001**
	No depression		10.80 ± 3.33		9.64 ± 5.17		3.88 ± 2.61	
Normal weight (N = 92)								
GAD-7 Interpretation		Anxiety	11.34 ± 3.44	0.844	11.09 ± 6.02	0.016	4.28 ± 3.05	0.214
		No anxiety	11.22 ± 3.56		7.92 ± 4.41		3.33 ± 2.31	
PHQ-9 Interpretation		Depression	11.10 ± 3.59	0.861	11.63 ± 6.17	**0.003**	4.66 ± 3.04	0.018
		No depression	11.34 ± 3.48		7.76 ± 4.19		3.18 ± 2.25	
Overweight (N = 254)								
GAD-7 Interpretation		Anxiety	10.76 ± 3.21	0.689	12.03 ± 6.01	**<0.001**	4.82 ± 2.79	**<0.001**
		No anxiety	10.90 ± 3.42		8.69 ± 5.16		3.57 ± 2.60	
PHQ-9 Interpretation		Depression	10.52 ± 3.56	0.233	11.70 ± 6.03	**<0.001**	4.77 ± 2.83	**0.001**
		No depression	11.09 ± 3.07		8.92 ± 5.27		3.59 ± 2.57	
Obesity I (N = 366)								
GAD-7 Interpretation		Anxiety	10.30 ± 3.42	0.183	12.63 ± 5.95	**<0.001**	5.11 ± 2.77	**<0.001**
		No anxiety	10.75 ± 3.28		10.13 ± 5.28		3.99 ± 2.63	
PHQ-9 Interpretation		Depression	10.04 ± 3.44	**0.007**	12.39 ± 6.18	**<0.001**	5.13 ± 2.64	**<0.001**
		No depression	11.02 ± 3.19		10.12 ± 5.03		3.88 ± 2.71	
Obesity II (N = 247)								
GAD-7 Interpretation		Anxiety	9.92 ± 3.44	0.566	13.01 ± 5.92	0.031	5.51 ± 0.26	0.018
		No anxiety	9.61 ± 3.42		11.39 ± 5.48		4.72 ± 2.64	
PHQ-9 Interpretation		Depression	9.78 ± 3.49	0.821	13.00 ± 6.01	0.028	5.42 ± 2.70	0.033
		No depression	9.77 ± 3.37		11.11 ± 5.16		4.72 ± 2.56	
Obesity III (N = 146)								
GAD-7 Interpretation		Anxiety	8.71 ± 3.96	0.084	13.78 ± 6.37	**0.002**	6.33 ± 4.35	**<0.001**
		No anxiety	9.89 ± 3.95		10.46 ± 5.49		4.35 ± 2.44	
PHQ-9 Interpretation		Depression	8.61 ± 3.91	0.016	13.94 ± 5.94	**<0.001**	6.28 ± 2.45	**<0.001**
		No depression	10.33 ± 3.91		9.48 ± 5.67		4.02 ± 2.47	

M—mean; SD—Standard deviation; N—number; GAD-7—Generalised anxiety disorder; PHQ-9—Patient Health Questionnaire-9; * Mann–Whitney U test Significant effects (<0.008) are marked in bold.

**Table 5:** Associations between studied variables and the TFEQ-R18 domain scores.

Variable		Cognitive Restraint		Uncontrolled Eating		Emotional Eating	
		M ± SD	*p*	M ± SD	*p*	M ± SD	*p*
BMI	Normal weight (N = 92)	11.26 ± 3.49	**<0.001 ^**	9.02 ± 5.22	**<0.001 ^**	3.66 ± 2.62	**<0.001 ^**
	Overweight (N = 254)	10.84 ± 3.30		10.14 ± 5.78		4.11 ± 2.75	
	Obesity I (N = 366)	10.56 ± 3.34		11.16 ± 5.69		4.45 ± 2.74	
	Obesity II (N = 247)	9.78 ± 3.43		12.28 ± 5.78		5.16 ± 2.66	
	Obesity III (N = 146)	9.21 ± 3.98		12.35 ± 6.21		5.48 ± 2.68	
Pharmacological treatment of obesity	Yes (N = 488)	10.96 ± 3.27	<0.001 *	11.30 ± 5.91	0.492 *	4.68 ± 2.77	0.387 *
	No (N = 617)	9.83 ± 3.60		11.04 ± 5.92		4.54 ± 2.76	
Semaglutide	Yes (N = 232)	11.19 ± 3.21	<0.001 *	10.78 ± 3.92	0.223 *	4.47 ± 2.70	0.441 *
	No (N = 873)	10.11 ± 3.54		11.25 ± 5.82		4.63 ± 2.77	
Liraglutide	Yes (N = 235)	10.84 ± 3.25	0.016 *	11.56 ± 5.41	0.125 *	4.69 ± 2.79	0.588 *
	No (N = 870)	10.20 ± 3.56		11.04 ± 5.96		4.58 ± 2.75	
Bupropion + Naltrexone	Yes (N = 41)	10.61 ± 3.71	0.401 *	12.98 ± 5.67	0.067 *	6.13 ± 2.39	<0.001 *
	No (N = 1064)	10.32 ± 3.51		11.08 ± 5.84		4.54 ± 2.75	
Surgical treatment of obesity	Yes (N = 131)	11.78 ± 3.57	<0.001 *	7.83 ± 5.35	<0.001 *	3.28 ± 2.54	<0.001 *
	No (N = 974)	10.14 ± 3.45		11.60 ± 5.77		4.77 ± 2.73	

M—mean; SD—Standard deviation; N—number; BMI—body mass index; kg/m^2^—kilogram(s) per square meter; ^ Kruskal–Wallis H Test; * Mann–Whitney U test; Significant effects (<0.017) are marked in bold.

**Table 6:** The analysis of the mediation effect of BMI in associations between the TFEQ-R18 subscales and anxiety (GAD-7) and health (PHQ-9).

Explained Variable	Factor	Effect Statistics	Coeff.	Lower Limit of 95% CI	Upper Limit of 95% CI	*p*
GAD-7 score	Cognitive Restraint	ACME	−0.03	−0.06	−0.01	**<0.001**
		ADE	−0.13	−0.22	−0.01	0.028
		Total effect	−0.16	−0.26	−0.04	0.016
		Prop. med. effect	0.20	0.28	163.79	0.016
	Uncontrolled Eating	ACME	0.01	0.00	0.02	**0.006**
		ADE	0.31	0.25	0.37	**<0.001**
		Total effect	0.33	0.27	0.38	**<0.001**
		Prop. med. effect	0.04	0.01	0.08	**0.006**
	Emotional Eating	ACME	0.03	0.01	0.06	0.026
		ADE	0.63	0.49	0.76	**<0.001**
		Total effect	0.66	0.52	0.79	**<0.001**
		Prop. med. effect	0.04	0.01	0.10	0.026
PHQ-9 score	Cognitive Restraint	ACME	−0.04	−0.07	−0.02	**<0.001**
		ADE	−0.25	−0.35	−0.13	**<0.001**
		Total effect	−0.29	−0.39	−0.18	**<0.001**
		Prop. med. effect	0.15	0.08	0.31	**<0.001**
	Uncontrolled Eating	ACME	0.02	0.01	0.03	**<0.001**
		ADE	0.38	0.32	0.44	**<0.001**
		Total effect	0.40	0.34	0.46	**<0.001**
		Prop. med. effect	0.05	0.02	0.09	**<0.001**
	Emotional Eating	ACME	0.05	0.02	0.08	**<0.001**
		ADE	0.72	0.59	0.86	**<0.001**
		Total effect	0.77	0.63	0.90	**<0.001**
		Prop. med. effect	0.06	0.03	0.12	**<0.001**

ACME—average causal mediation effect; ADE—average direct effect; Prop. Med. Effect—proportion of mediated effect (ACME/Total effect); GAD-7—Generalized anxiety disorder; PHQ-9—Patient Health Questionnaire-9; Significant effects (<0.008) are marked in bold.

## Data Availability

The data presented in this study are available on request from the corresponding author. The data are not publicly available due to lack of a suitable platform.

## Supplementary Materials

The following supporting information can be downloaded at: https://www.mdpi.com/article/10.3390/nu16010080/s1. Table S1: The English Version of the Study Questionnaire and the distribution of responses among participants in the TFEQ-R18, GAD-7, and PHQ-9 questionnaires; Table S2: The distribution of the responses among the participants in the Three Factor Eating Questionnaire-18 (TFEQ-R18); Table S3: The distribution of the responses among the participants in the Patient Health Questionnaire-9 (PHQ-9); and Table S4: The distribution of the responses among the participants in the General Anxiety Disorder-7 Questionnaire (GAD-7).
