# Supplementary material for: Maladaptive Eating Behaviours, Generalised Anxiety Disorder and Depression Severity: A Comparative Study between Adult Women with Overweight, Obesity, and Normal Body Mass Index Range
Source: Nutrients. 2023 Dec 26;16(1):80. doi: 10.3390/nu16010080 (PMC10780963; doi:10.3390/nu16010080)
Supplement: Supplementary file 1 [file nutrients-16-00080-s001.zip › nutrients-2761865-supplementary.pdf]

Table S1. The English Version of the Study Questionnaire and the distribution of responses among participants in TFEQ-R18, GAD-7 and PHQ-9 questionnaires.

**Demographic variables**	
**How old are you? [years]**	
**Sex**	Female
	Male
	Other
**What is your height? [in centimeters]**	
**What is your weight? [in kilograms]**	
**Is this currently your highest body weight in your life? For women, if applicable, please exclude weight from pregnancy and postpartum period (6 weeks after childbirth).**	Yes
	No
**Additional question after choosing „No" in Q: Is this currently your highest body weight in your life?**	
**What was your highest body weight in your life? [in kilograms]**	
**Are you currently using any of the listed medications for treating obesity? (multiple choice)**	Saxenda or Victoza (active ingredient liraglutide)
	Ozempic or Wegovy (active ingredient semaglutide)
	Mysimba (active ingredient Bupropion Hydrochloride + Naltrexone Hydrochloride)
	I don't use any of the above
**Have you undergone bariatric surgery in the past?**	Yes
	No
**Additional question after choosing „Yes" in Q: Have you undergone bariatric surgery in the past?**	
**What kind of procedure did you have?**	Gastric Sleeve surgery
	Gastric Bypass surgery
	Gastric Band surgery
	Endoscopic intragastric balloon
	Other
**Do you have any chronic illnesses?**	Yes

	No
**Additional question after choosing „Yes" in Q: Do you have any chronic illnesses?**	
**What chronic illness do you have? (multiple choice)**	Obesity
	Hypertension
	Cardiovascular disease other than hypertension
	Diabetes Mellitus type 2
	Arthritis
	Hypothyroidism
	Depressive Disorder
	General Anxiety Disorder
	Dyslipidemia
	Hepatic Steatosis
	Other, not mentioned above
**Three Factor Eating-18 Questionnaire (TFEQ-R18)**	
**I deliberately take small helpings as a means of controlling my weight.**	Definitely true
	Mostly true
	Mostly False
	Definitely False
**When I feel anxious, I find myself eating.**	Definitely true
	Mostly true
	Mostly False
	Definitely False
**Being with someone who is eating often makes me hungry enough to eat also.**	Definitely true
	Mostly true
	Mostly False
	Definitely False
**When I feel sad, I find myself overeating.**	Definitely true
	Mostly true
	Mostly False
	Definitely False
**When I see a real delicacy, I often get so hungry that I have to eat right away.**	Definitely true
	Mostly true

	Mostly False
	Definitely False
**I get so hungry that my stomach often seems like a bottomless pit.**	Definitely true
	Mostly true
	Mostly False
	Definitely False
**I am always hungry so it is hard for me to stop eating before I finish the food on my plate.**	Definitely true
	Mostly true
	Mostly False
	Definitely False
**When I feel lonely, I console myself by eating.**	Definitely true
	Mostly true
	Mostly False
	Definitely False
**I deliberately take small helpings as a means of controlling my weight.**	Definitely true
	Mostly true
	Mostly False
	Definitely False
**I do not eat some foods because they make me fat.**	Definitely true
	Mostly true
	Mostly False
	Definitely False
**I am always hungry enough to eat at any time.**	Definitely true
	Mostly true
	Mostly False
	Definitely False
**When I smell a sizzling steak or juicy meatloaf I find it very difficult to keep from eating, even if I have just finished a meal.**	Definitely true
	Mostly true
	Mostly False
	Definitely False
**Sometimes when I start eating, I just can't seem to stop.**	Definitely true
	Mostly true

	Mostly False
	Definitely False
**How often do you feel hungry?**	Only at meal times
	Sometimes between meals
	Often between meals
	Almost always
**How likely are you to consciously eat less than you want?**	Unlikely
	Slightly likely
	Moderately likely
	Very likely
**How frequently do you avoid buying tempting foods?**	Almost never
	Seldom
	Moderately likely
	Almost always
**Do you go on eating binges though you are not hungry?**	Never
	Rarely
	Sometimes
	At least once a week
**How much do you restrict your food intake? Mark on a scale from 1 to 8.**	1 (never restricting)
	2
	3
	4
	5
	6
	7
	8 (always restricting)
**General Anxiety Disorder-7 Questionnaire (GAD-7)**<br>**Over the last two weeks, how often have you been bothered by the following problems?**	
**Feeling nervous, anxious, or on edge.**	Not at all
	Several days
	More than half the days
	Nearly every day

**Not being able to stop or control worrying.**	Not at all
	Several days
	More than half the days
	Nearly every day
**Worrying too much about different things.**	Not at all
	Several days
	More than half the days
	Nearly every day
**Trouble relaxing.**	Not at all
	Several days
	More than half the days
	Nearly every day
**Being so restless that it is hard to sit still.**	Not at all
	Several days
	More than half the days
	Nearly every day
**Becoming easily annoyed or irritable.**	Not at all
	Several days
	More than half the days
	Nearly every day
**Feeling afraid, as if something awful might happen.**	Not at all
	Several days
	More than half the days
	Nearly every day
**Patients Health Questionnaire-9 (PHQ-9)<br>Over the last 2 weeks, how often have you been bothered by any of the following problems?**	
**Little interest or pleasure in doing things.**	Not at all
	Several days
	More than half the days
	Nearly every day
**Feeling down, depressed, or hopeless.**	Not at all
	Several days

	More than half the days
	Nearly every day
**Trouble falling or staying asleep, or sleeping too much.**	Not at all
	Several days
	More than half the days
	Nearly every day
**Feeling tired or having little energy.**	Not at all
	Several days
	More than half the days
	Nearly every day
**Poor appetite or overeating.**	Not at all
	Several days
	More than half the days
	Nearly every day
**Feeling bad about yourself — or that you are a failure or have let yourself or your family down.**	Not at all
	Several days
	More than half the days
	Nearly every day
**Trouble concentrating on things, such as reading the newspaper or watching television.**	Not at all
	Several days
	More than half the days
	Nearly every day
**Moving or speaking so slowly that other people could have noticed? Or the opposite — being so fidgety or restless that you have been moving around a lot more than usual**	Not at all
	Several days
	More than half the days
	Nearly every day
**Thoughts that you would be better off dead or of hurting yourself in some way.**	Not at all
	Several days
	More than half the days
	Nearly every day

Table S2. The distribution of the responses among the participants in the Three Factor Eating Questionnaire-18 (TFEQ-R18).

**Three Factor Eating-18 Questionnaire (TFEQ-R18)**		**n (n%)**
**I deliberately take small helpings as a means of controlling my weight.**	Definitely true	217 (19.6)
	Mostly true	427 (38.6)
	Mostly False	347 (41.7)
	Definitely False	114 (10.3)
**When I feel anxious, I find myself eating.**	Definitely true	281 (25.4)
	Mostly true	305 (27.6)
	Mostly False	345 (31.2)
	Definitely False	174 (15.7)
**Being with someone who is eating often makes me hungry enough to eat also.**	Definitely true	147 (13.3)
	Mostly true	318 (28.8)
	Mostly False	459 (41.5)
	Definitely False	181 (16.4)
**When I feel sad, I find myself overeating.**	Definitely true	236 (21.4)
	Mostly true	321 (29)
	Mostly False	372 (33.7)
	Definitely False	176 (15.9)
**When I see a real delicacy, I often get so hungry that I have to eat right away.**	Definitely true	173 (15.7)
	Mostly true	407 (36.8)
	Mostly False	425 (38.5)
	Definitely False	100 (9)

**I get so hungry that my stomach often seems like a bottomless pit.**	Definitely true	167 (15.1)
	Mostly true	230 (20.8)
	Mostly False	476 (43.1)
	Definitely False	232 (21)
**I am always hungry so it is hard for me to stop eating before I finish the food on my plate.**	Definitely true	106 (9.6)
	Mostly true	189 (17.1)
	Mostly False	487 (44.1)
	Definitely False	323 (29.2)
**When I feel lonely, I console myself by eating.**	Definitely true	211 (19.1)
	Mostly true	286 (25.9)
	Mostly False	358 (32.4)
	Definitely False	250 (22.6)
**I deliberately take small helpings as a means of controlling my weight.**	Definitely true	191 (17.3)
	Mostly true	482 (43.6)
	Mostly False	346 (31.3)
	Definitely False	86 (7.8)
**I do not eat some foods because they make me fat.**	Definitely true	311 (28.1)
	Mostly true	381 (34.5)
	Mostly False	315 (28.5)
	Definitely False	98 (8.9)
**I am always hungry enough to eat at any time.**	Definitely true	84 (7.6)
	Mostly true	247 (22.4)
	Mostly False	518 (46.9)
	Definitely False	256 (23.2)

**When I smell a sizzling steak or juicy meatloaf I find it very difficult to keep from eating, even if I have just finished a meal.**	Definitely true	67 (6.1)
	Mostly true	159 (14.4)
	Mostly False	503 (45.5)
	Definitely False	376 (34)
**Sometimes when I start eating, I just can't seem to stop.**	Definitely true	131 (11.9)
	Mostly true	248 (22.4)
	Mostly False	414 (37.5)
	Definitely False	312 (28.2)
**How often do you feel hungry?**	Only at meal times	191 (17.3)
	Sometimes between meals	599 (54.2)
	Often between meals	259 (23.4)
	Almost always	56 (5.1)
**How likely are you to consciously eat less than you want?**	Unlikely	31 (2.8)
	Slightly likely	378 (34.2)
	Moderately likely	538 (48.7)
	Very likely	158 (14.3)
**How frequently do you avoid buying tempting foods?**	Almost never	45 (4.1)
	Seldom	290 (26.2)
	Moderately likely	681 (61.6)
	Almost always	89 (8.1%)
**Do you go on eating binges though you are not hungry?**	Never	203 (18.4)
	Rarely	379 (34.3)
	Sometimes	409 (37)
	At least once a week	114 (10.3)

**How much do you restrict your food intake? Mark on a scale from 1 to 8.**	1 (never restricting)	115 (10.4)
	2	
	3	305 (27.6)
	4	
	5	532 (48.1)
	6	
	7	153 (13.8)
	8 (always restricting)	

Table S3. The distribution of the responses among the participants in the Patients Health Questionnaire-9 (PHQ-9)

**Patients Health Questionnaire-9 (PHQ-9) Over the last 2 weeks, how often have you been bothered by any of the following problems?**		**n (n%)**
**Little interest or pleasure in doing things.**	Not at all	275 (24.9)
	Several days	460 (41.6)
	More than half the days	225 (20.4)
	Nearly every day	145 (13.1)
**Feeling down, depressed, or hopeless.**	Not at all	297 (26.9)
	Several days	463 (41.9)
	More than half the days	173 (15.7)
	Nearly every day	172 (15.6)
**Trouble falling or staying asleep, or sleeping too much.**	Not at all	226 (20.5)
	Several days	342 (31)
	More than half the days	200 (18.1)

	Nearly every day	337 (30.5)
**Feeling tired or having little energy.**	Not at all	89 (8.1)
	Several days	327 (29.6)
	More than half the days	269 (24.3)
	Nearly every day	420 (38)
**Poor appetite or overeating.**	Not at all	290 (26.2)
	Several days	417 (37.7)
	More than half the days	232 (21)
	Nearly every day	166 (15)
**Feeling bad about yourself — or that you are a failure or have let yourself or your family down.**	Not at all	334 (30.2)
	Several days	322 (29.1)
	More than half the days	192 (17.4)
	Nearly every day	257 (23.3)
**Trouble concentrating on things, such as reading the newspaper or watching television.**	Not at all	393 (35.6)
	Several days	321 (29)
	More than half the days	202 (18.3)
	Nearly every day	189 (17.1)
**Moving or speaking so slowly that other people could have noticed? Or the opposite — being so fidgety or restless that you have been moving around a lot more than usual**	Not at all	707 (64)
	Several days	229 (20.7)
	More than half the days	107 (9.7)

	Nearly every day	62 (5.6)
Thoughts that you would be better off dead or of hurting yourself in some way.	Not at all	856 (77.5)
	Several days	152 (13.8)
	More than half the days	40 (3.6)
	Nearly every day	57 (5.2)

Table S4. The distribution of the responses among the participants in the General Anxiety Disorder-7 Questionnaire (GAD-7)

**General Anxiety Disorder-7 Questionnaire (GAD-7) Over the last two weeks, how often have you been bothered by the following problems?**		**n (n%)**
**Feeling nervous, anxious, or on edge.**	Not at all	186 (16.8)
	Several days	407 (36.8)
	More than half the days	216 (19.6)
	Nearly every day	296 (26.8)
**Not being able to stop or control worrying.**	Not at all	263 (23.8)
	Several days	414 (37.5)
	More than half the days	201 (18.2)
	Nearly every day	227 (20.5)
**Worrying too much about different things.**	Not at all	188 (17)
	Several days	387 (35)
	More than half the days	237 (21.4)
	Nearly every day	293 (26.5)
**Trouble relaxing.**	Not at all	215 (19.5)
	Several days	332 (30)
	More than half the days	259 (23.4)

	Nearly every day	299 (27.1)
**Being so restless that it is hard to sit still.**	Not at all	466 (42.2)
	Several days	351 (31.8)
	More than half the days	182 (16.5)
	Nearly every day	106 (9.6)
**Becoming easily annoyed or irritable.**	Not at all	195 (17.6)
	Several days	407 (36.8)
	More than half the days	231 (20.9)
	Nearly every day	272 (24.6)
**Feeling afraid, as if something awful might happen.**	Not at all	453 (41)
	Several days	325 (29.4)
	More than half the days	157 (14.2)
	Nearly every day	170 (15.4)
